# IL4I1 Accelerates the Expansion of Effector CD8^+^ T Cells at the Expense of Memory Precursors by Increasing the Threshold of T-Cell Activation

**DOI:** 10.3389/fimmu.2020.600012

**Published:** 2020-12-04

**Authors:** Marie-Line Puiffe, Aurélie Dupont, Nouhoum Sako, Jérôme Gatineau, José L. Cohen, Denis Mestivier, Agnès Lebon, Armelle Prévost-Blondel, Flavia Castellano, Valérie Molinier-Frenkel

**Affiliations:** ^1^ Virus-Immunity-Cancer Department, Institut Mondor de Recherche Biomédicale (IMRB), INSERM U955, Université Paris-Est Créteil, Créteil, France; ^2^ Bioinformatics Core Laboratory, Institut Mondor de Recherche Biomédicale (IMRB), INSERM U955, Université Paris-Est Créteil, Créteil, France; ^3^ INSERM U1016, CNRS UMR8104, Institut Cochin, Université de Paris, Paris, France; ^4^ Pathobiology Department, Groupe Hospitalo-Universitaire Chenevier-Mondor, AP-HP, Créteil, France

**Keywords:** immunosuppressive enzyme, T cell immune response, CD8 T cell, viral infection, T-cell activation, T-cell priming, lymphocytic choriomeningitis virus, memory precursor cells

## Abstract

IL4I1 is an immunoregulatory enzyme that inhibits CD8 T-cell proliferation *in vitro* and in the tumoral context. Here, we dissected the effect of IL4I1 on CD8 T-cell priming by studying the differentiation of a transgenic CD8 T-cell clone and the endogenous repertoire in a mouse model of acute lymphocytic choriomeningitis virus (LCMV) infection. Unexpectedly, we show that IL4I1 accelerates the expansion of functional effector CD8 T cells during the first several days after infection and increases the average affinity of the elicited repertoire, supporting more efficient LCMV clearance in WT mice than IL4I1-deficient mice. Conversely, IL4I1 restrains the differentiation of CD8 T-cells into long-lived memory precursors and favors the memory response to the most immunodominant peptides. IL4I1 expression does not affect the phenotype or antigen-presenting functions of dendritic cells (DCs), but directly reduces the stability of T-DC immune synapses *in vitro*, thus dampening T-cell activation. Overall, our results support a model in which IL4I1 increases the threshold of T-cell activation, indirectly promoting the priming of high-affinity clones while limiting memory T-cell differentiation.

## Introduction

The negative control of T-cell responses is regulated by multiple factors, including amino-acid catabolizing enzymes. These enzymes regulate the availability of essential or semi-essential amino acids in the proximal environment of T cells and produce bioactive metabolites with proapoptotic or antiproliferative effects. IL4-induced gene 1 (IL4I1) is an L-phenylalanine oxidase that produces phenylpyruvate, hydrogen peroxide, and ammonia (1). It limits the activation and proliferation of T cells and modulates CD4 T-cell differentiation, at least partially, through its enzymatic activity. IL4I1 is focally secreted by antigen-presenting cells in the synaptic cleft and attaches to the T cell (2). It is mainly produced by macrophages and dendritic cells (DCs) in the context of pro-T helper type 1 (Th1) inflammatory stimuli (3). IL4I1 is particularly abundant in the tumor microenvironment of inflammatory human cancers and may be associated with a poor prognosis [reviewed in (4)]. Indeed, murine models have shown that IL4I1 negatively regulates the expansion and functionality of antitumor CD8^+^ T cells (5, 6), an effect that has been confirmed by the analysis of human primary cutaneous melanomas (7).

The continuous provision of cytotoxic CD8^+^ effectors depends on the self-renewal capacity and multipotency of long-lived memory cells (8). Several types of CD8^+^ memory T-cells have been described, from stem cell-like to circulating effector-memory or tissue-resident cells (9). The characterization of these subsets is complicated by the considerable heterogeneity and plasticity of specific T cells during the days and weeks that follow an antigenic challenge, as the cells dynamically regulate their phenotype, functions, “stemness,” and recirculation patterns (10). However, certain markers, such as killer cell lectin-like receptor G1 (KLRG1) and the IL-7 receptor (CD127) can help in the early prediction of the differentiation fate of the antigen-specific population (11, 12), with CD127^-^KLRG1^hi^ cells corresponding to terminally-differentiated short-lived effector cells, whereas CD127^+^ cells preferentially give rise to recirculating central memory cells. Two principal experimentally-supported theories have been proposed for the formation of memory cells: early segregation of discrete precursors and/or progressive differentiation from effector T cells (13, 14). Regardless of the model, it is recognized that signals received during the priming phase are strong determinants of the fate of the specific T-cell population that persists after antigen elimination.

Acute infection by a slowly-replicating lymphocytic choriomeningitis virus (LCMV) strain is a widely used mouse model for studying CD8^+^ T-cell priming that makes it possible to divide the CD8^+^ T-cell response into three successive well-defined phases (15). Following viral challenge, DCs present immunodominant peptides and the resulting inflammation helps to induce vigorous expansion of CD8^+^ T cells, which reaches a peak at the time (around day 8) of resolution of the infection. Terminally-differentiated effector T cells are then eliminated during a 2-week contraction phase, allowing the progressive emergence of memory cells (16–18).

We compared the CD8^+^ T-cell response to LCMV in mice with (WT) or without (IL4I1^-/-^) IL4I1 to decipher the role of IL4I1 during the first steps of CD8^+^ T-cell differentiation. Unexpectedly, we observed accelerated priming of CD8^+^ T cells and early differentiation of functional effector T cells in WT mice relative to IL4I1^-/-^ mice. We excluded variations in IFNα production and the modification of DC subsets. *In vitro* assays on DC-T cell co-cultures confirmed a direct inhibitory effect of IL4I1 on CD8^+^ T-cell activation that is mitigated by clonal competition between T cells. The analysis of responding CD8^+^ T cells in infected IL4I1^-/-^ mice showed the repertoire of LCMV-specific CD8^+^ T cells to be of lower affinity than those in WT mice and that CD8^+^ T cells preferentially differentiate into memory precursor cells. Thus, IL4I1 may directly restrain the priming of low-affinity CD8^+^ T cells by enhancing the activation threshold.

## Materials and Methods

### Cell Culture, Reagents, and Peptides

Cell culture was performed in RPMI supplemented with 10% fetal calf serum, 50 µM β-mercaptoethanol, penicillin (100 UI/ml), and streptomycin (100 mg/ml). Cell culture reagents were purchased from Thermo Fisher Scientific (France), except for fetal calf serum (Biowest). Collagenase IV S was purchased from Sigma-Aldrich, DNase I from Roche, ovalbumin from Interchim (France), and 3,3’,5,5’-Tetramethylbenzidine from Mabtech. LCMV peptides GP33 (KAVYNFATC), NP366 (FQPQNGQFI), GP276 (SGVENPGGYCL), NP205 (YTVKYPNL), and influenza peptide NP50 (ASNENMDAM) were purchased from MBL international.

### Mice and Virus

C57BL/6J WT mice were purchased from Janvier Labs (Le Genest-Saint-Isle, France). Mice deficient for the IL4I1 gene (IL4I1^-/-^) were purchased from the Texas A&M Institute for Genomic Medicine, backcrossed onto a full C57BL/6J genetic background, and maintained at the TAAM (Orleans, France). Rag2^-/-^ mice expressing the CD45.1 allotype marker and a monoclonal Vα2/Vβ8.1 TCR specific for H-2D^b^GP33 complexes, backcrossed onto a full C57BL/6 genetic background (19), were kindly given by Dr Benedita Rocha and maintained in the TAAM facility. All animals were housed under specific pathogen-free conditions. Mouse experiments were carried out in accordance with the guidelines of the French Veterinary Department and were approved by the local Ethical Committee for Animal Experimentation (Cometh Anses/ENVA/UPEC) and the French Research ministry under the number 05338.03.

LCMV clone WE2.2 (10^5^ PFU per mouse) was injected into the retro-orbital sinus at day 0. The titers of the LCMV stocks and those of the infectious virus in the mouse spleens were determined by plaque assay on Vero cells. For adoptive transfer experiments, 5,000 CD8^+^ T cells were sorted from P14 mouse spleens and transferred i.v. at day 1.

### Cell Sorting

CD8^+^ T cells from P14 or WT mice were magnetically sorted from spleens after mechanical dissociation and red blood cell lysis using the EasySep mouse CD8^+^ T-cell enrichment kit (Stemcell). Purified T cells were maintained overnight in complete medium at a concentration of 1 x 10^6^ cells/ml before starting coculture with DCs. Purity was assessed by flow cytometry and was > 90%.

For DC purification, spleens were mechanically dissociated and treated with 16 mg/ml collagenase and 10 mg/ml DNAse at 37°C for 30 min and then filtered through a 40-µm cell strainer. Negative cell sorting was performed using the mouse PanDC Enrichment kit (Miltenyi Biotec) according to the manufacturer’s instructions. DCs were conserved overnight in PBS, 1% FCS, and 2 mM EDTA at 4°C before use. Purity of the DC cell suspension was assessed by flow cytometry and reached 70% for the transcriptomic experiments.

### Flow Cytometry

GP33-specific CD8^+^ lymphocytes were labeled with PE-coupled H2-D^b^GP33 tetramers from MBL international. Fc receptors were blocked with anti-CD16/32 mAbs (clone 2.4G2, BD Biosciences). Dead cells were excluded using Fixable Viability Dye eFluor^®^ 450 or 780 (eBioscience).

T cells were analyzed using the appropriate combinations of the following antibodies: APC-anti-CD44 (clone KM201) from SouthernBioTech, Alexa Fluor 647-anti-Mouse Ki-67 (clone B56), FITC-anti-Vβ8.1/8.2 (clone MR5-2) from BD PharMingen, PEcy7-anti-CD45.1 (clone A20) and -CD45.2 (clone 104) from BioLegend, APC-anti-CD44 (clone IM7), BV605-anti-CD25 (clone PC61) PerCP-eFluor710- or BV605-anti-KLRG1 (clone 2F1), FITC- or BV421-anti-CD127 (Clone HIL-7R-M21) from BD Bioscience, FITC-anti-granzyme B (clone REA226) from Miltenyi, and CD69-FITC (Clone H1.2F3), APCeFluor780-anti-CD8α (clone 53-6,7), FITC-anti-Bcl2 (clone 10C4), PE-anti-T-Bet (clone eBio4B10), PerCPeFluor710-anti-Eomes (clone Da11mag), PerCPeFluor71-anti-TNFα (clone MP6-XT22), and APC-anti-INF*γ* (clone XMG1.2), all from eBioscience.

DC subsets were analyzed using the following antibodies: BB515-anti-CD11b (clone MI/70) and BV605-anti-CD11c (clone HL3) from BD Biosciences, APC-anti-CD317 (clone eBio927), APCeFluor780-anti-CD8α (clone 53-6.7) from eBioscience, and PE-anti-SIGLEC-H (clone 551.3D3) from Miltenyi. Lineage cell exclusion was conducted using BV510-coupled anti-NK1.1 (clone PK136), CD19 (clone 1D3) and CD3 (clone 17A2) from BD Biosciences. DC maturation was analyzed using PECF594-anti-CD40 (clone 3/23), PEcy7-anti-CD86 (clone GL1), BV711-anti-CD80 (clone 16-10A1), and BV421-anti-IA^b^ (clone AF6-120.1), all from BD Biosciences.

Isotype controls recommended by the manufacturer were used in FMO controls. Antibody incubations were performed at 4°C for 30 min in PBS with 1% human AB serum. For the analysis of circulating cells, red blood cells were lysed after staining using VersaLyse Lysing Solution (Beckman-Coulter). For intracellular staining, cells were fixed and permeabilized using the FoxP3 Fixation/Permeabilization kit (eBioscience) or Cytofix/Cytoperm (BD Bioscience). Cell acquisition was performed on an LSRFortessa (BD) and the data analyzed using FlowJo software.

### ELISPOT-IFN*γ*

PVDF plates (Millipore) were EtOH treated and coated with anti-mIFN*γ* (clone AN-18) before adding splenocytes (days 6, 8, and 12 p.i.: 5 x 10^4^/well; ≥ day 35: 10^5^/well) in complete medium with increasing quantities of the stimulating peptides for 16 to 18 h at 37°C in 5% CO_2_. Revelation was performed using anti-mIFN*γ* antibodies conjugated to biotin (clone R4-6A2 from Mabtech) for 2 h at RT, followed by streptavidin-horseradish peroxidase-conjugated antibodies (Mabtech) for 1 h at RT and a 15-min revelation with 3,3’,5,5’-tetramethylbenzidine. ELISPOT assays were performed in triplicate and the spots counted by an automatic ELISPOT reader (Autoimmun Diagnostika GmbH).

### Antigen Processing by DCs

Isolated splenocytes were resuspended at 1 x 10^6^ cells/ml in complete medium supplemented or not with 5 µg/ml ovalbumin for 2 h at 37°C before staining. Ovalbumin processing was assessed by staining splenocytes with the K^b^OVA-PE antibody from eBioscience (Clone eBio25-D1.16), which recognizes the OVA257 peptide (SIINFEKL) bound to H-2K^b^, together with anti-CD11b-BB515 (Clone MI/70), anti CD11c- BV605 (Clone HL3), and Fixable Viability Stain 510, all from BD Biosciences.

### Transcriptome Analysis

Total RNA was isolated from purified DCs using RLT buffer and RNeasy columns (Qiagen). Libraries of polyA mRNA were generated using the TruSeq^®^ Stranded mRNA Library Prep kit (Illumina) with double indexing using TruSeq RNA UD Illumina Indexes. RNA was reverse transcribed using SuperScript™ II (Invitrogen). Next-generation sequencing was performed by 75 bp single reading with the NextSeq 500/550 High Output Kit v2.5 (75 Cycles) on a NextSeq 500 analyzer (all from Illumina). The six samples (18,446,620 ± 1,924,912 reads/sample, single end, 75 pb) were quality-checked using the software FastQC (version v0.11.9). We checked that rRNA depletion was of the expected quality (less than 2.03 ± 0.17% of total RNA, no prokaryotic contamination) and that more than 98% of the reads mapped to the mouse genome (GRCm38) using SortMeRNA (version 2.1), Fastq-screen (version 0.14.0) and Kraken2 (version 2.0.8/default database). Trimmomatic (version 0.39) was used to filter reads using a quality of 20 (sliding window of 5 reads) and a minimal length of 50 pb, which led to more than 98% of surviving reads. Filtered reads (17,584,772 ± 1.846.171 reads/sample) were aligned to the mouse genome (GRCm38) using STAR (version 2.6.1d) and the level of gene expression calculated using RSEM (version v1.3.1). Differentially expressed genes were assessed using EdgeR (version 3.28.1) for genes with an abundance > 1 CPM (counts per million) in at least four samples. Heatmaps were generated using the pheatmap library (version 1.0.12). For Gene Ontology comparisons, we generated a file of every GO term for each gene using in house Python scripts that allowed us to focus on specific pathways (complete list of GO terms in **Figure S6**).

### Cocultures of DCs With P14 CD8^+^ T Cells

Sorted DCs (10^6^ cells/ml) were pulsed with GP33 (0.1 µg/ml final concentration) for 1 h at 37°C in 5% CO_2_, washed, and resuspended at 10^5^ cells/ml in complete medium. DCs were then cultured with sorted P14 CD8^+^ T cells in round-bottom 96-well plates in a 5% CO_2_, 37°C incubator at a ratio of 1 DC per 10 P14 cells. In certain experiments, P14 T cells were stained with Cell Proliferation Dye (CPD) eFluor™ 670. Recombinant murine IL4I1 (R&D systems) was used at 100 ng/ml in cocultures.

For the detection of DC-T cell conjugates, DCs were stained with CellTrace CFSE, or CellTrace CFSE (WT DCs) and CellTrace Violet (IL4I1^-/-^ DCs). CPD and CellTrace reagents (all from eBioscience) were used according to the manufacturer’s protocol. DCs (2 x 10^6^ cells/ml) were pulsed for 1 h with GP33 or NP50 (0.5 µg/ml in complete medium). DCs were stained with two different tracers were mixed in equal amounts. P14 T cells stained with CPD were resuspended at 2 x 10^6^cells/ml in complete medium. DC-P14 T cell co-cultures (ratio 1:10) were set up in 5-ml round-bottom polystyrene tubes for 30 min to 24 h (37°C, 5% CO_2_). Cells were then fixed by gently adding an equal volume of a 2% PFA solution and incubating for 20 min. Results were acquired immediately after fixation. For experiments in which P14 T cells were mixed with naïve polyclonal CD8^+^ T cells, one P14 cell was added to four polyclonal T cells. The ratio of WT or IL4I1^-/-^ DCs to P14 T cells was maintained at 1:10 for all conditions tested.

### Cytokine Quantification

IFNα was quantified in plasma by ELISA (R&D systems). IP10, IL-6, IL-12p70, TNFα, GM-CSF, and IL-1β were quantified in plasma by Meso Scale Discovery (MSD) multiplex immunoassay. IL-2 was quantified by ELISA in the medium supernatant (BD Biosciences). All kits were used according to the manufacturer’s instructions.

### Statistics

Values are expressed as the mean ± SEM where applicable. All statistical analyses were performed using Prism 7 software (GraphPad). The tests used are indicated in the figure legends. Data from two independent groups were compared using paired or unpaired t tests, depending on the inter-experiment variation of the values. The unpaired Student t test was used if values followed a normal distribution and the Mann-Whitney test otherwise. For more than two simultaneous comparisons, a two-way ANOVA was used, combined with Sidak’s post-test in **Figure 7E**. *p < 0.05, **p < 0.01, ***p < 0.001, and ****p < 0.0001.

## Results

### IL4I1 Accelerates the Development of Functional Effector T Cells Against LCMV Immunodominant Epitopes

**Figure 1:**
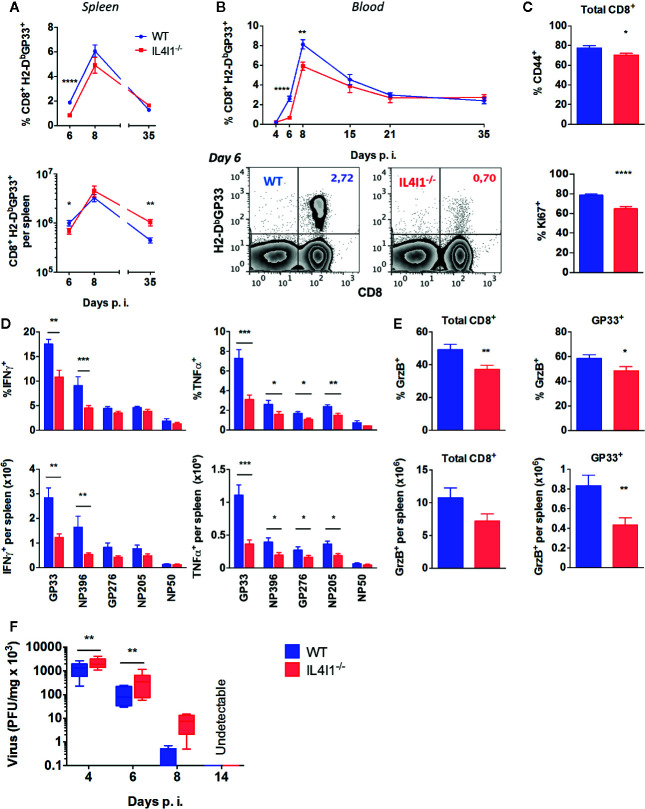
Reduced number of functional effector CD8 T cells in LCMV-infected IL4I1^-/-^mice. WT (blue) and IL4I1^-/-^ (red) mice were infected i.v. with LCMV on day 0 **(A, B)**. The post-infection (p.i.) percentage of GP33-specific CD8 T cells among viable lymphocytes was measured in the spleen (**A**, upper graph) and blood **(B)** by flow cytometry using H2-D^b^GP33 tetramers. Total numbers of splenic GP33-specific CD8 T cells were calculated based on viable splenocyte counts (**A**, lower graph). Representative data in the blood at day 6 are shown in **(B)**, lower panels **(C)**. Expression of the activation marker CD44 (upper graph) and of the mitotic marker Ki67 (lower graph) by splenic CD8^+^ T lymphocytes at day 6 **(D)**. Intracellular IFN*γ* and TNFα at day 6, after *in vitro* stimulation of splenocytes with the LCMV peptides GP33, NP396, GP376, and NP205 and control influenza peptide NP50. The upper graphs show the percentage of CD44^+^ CD8^+^ T cells and the lower graphs the calculated total number in the spleen **(E)**. Intracellular granzyme B (GzB) at day 6. The upper graphs show the percentage of total and GP33-specific CD44^+^ CD8^+^ T cells and the lower graphs the calculated total number in the spleen **(A–E)**. The means ± SEM from three to four independent experiments with three to four mice per group are shown **(F)**. Viral titers were measured in the spleen (PFU, plaque-forming units). Mean ± SEM from two (day 14) to 11 (day 4) independent experiments with one to two mice per group. *p < 0.05, **p < 0.005, ***p < 0.001; ****p < 0.0001 (Student t test: **A–E**; Wilcoxon paired t test: **F**).

We evaluated the role of IL4I1 in the early steps of the CD8^+^ T-cell response using the LCMV acute resolutive infection model. We first measured the kinetics of CD8^+^ T cells that recognize the immunodominant GP33 epitope in the spleen (**Figure 1A**; upper panel, percentages; lower panel, absolute counts) and blood (**Figure 1B**) from the expansion phase to the beginning of the memory phase. Far fewer GP33-specific CD8^+^ T cells were detected in IL4I1^-/-^ mice at day 6 than in WT mice, both in the blood and spleen. At the peak of the response (day 8), this difference was still perceivable in the blood. At the end of the contraction phase (day 15), GP33-reactive T cells reached similar percentages in WT and IL4I1^-/-^ mice (**Figure 1B**), but the absolute counts were, on average, 64% higher in the spleens of IL4I1^-/-^ mice at the beginning of the memory phase (day 35; **Figure 1A** lower panel). At day 6, the decreased expansion of GP33-specific T cells in IL4I1^-/-^ mice was associated with lower activation and proliferation of CD8^+^ T cells (**Figure 1C**).

We next explored the effector functions of LCMV-specific T cells in the spleens of the two strains during the expansion phase, day 6 post infection. Both the proportion and absolute number of GP33-specific T cells secreting IFNγ and TNFα were lower in IL4I1^-/-^ than WT mice (**Figure 1D** **)**. CD8^+^ T cells from IL4I1^-/-^ mice also produced less IFNγ and/or TNFα in response to three other LCMV immunodominant peptides (NP396, GP276, NP205; **Figure 1D**). Finally, there were both fewer granzyme B-producing GP33-specific and total CD8^+^ T cells in IL4I1^-/-^ than WT mice (**Figure 1E**). Accordingly, elimination of the virus in the spleens was slightly delayed in IL4I1^-/-^ mice, but infectious virus was no longer detectable in either strain at day 14 (**Figure 1F**).

Thus, the development of the cytotoxic T-cell response to LCMV is slower in IL4I1^-/-^ than WT mice. These results are counterintuitive, as IL4I1 limits TCR activation and T-cell proliferation.

### IL4I1 Diminishes the Differentiation of Memory Precursor CD8^+^ T Cells

**Figure 2:**
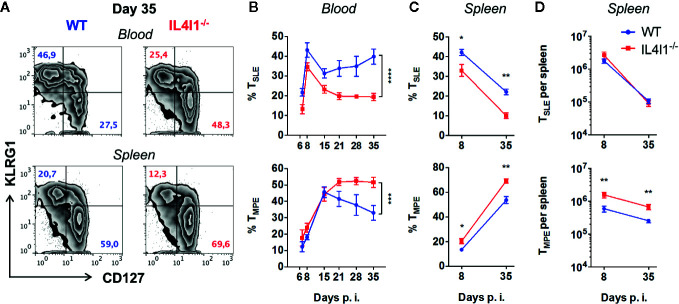
Diminution of GP33-specific T_SLE_ with a reciprocal increase of T_MPE_ in LCMV-infected IL4I1^-/-^ mice. WT (blue) and IL4I1^-/-^ (red) mice were infected i.v. with LCMV on day 0. H2-D^b^GP33^+^ CD8^+^ T lymphocytes were analyzed for KLRG1 and CD127 expression by flow cytometry at various times p.i. **(A)** Representative data obtained at day 35 for the blood (upper panels) and spleen (lower panels). **(B)** Percentage of circulating CD127^-^ KLRG1^+^ T_SLE_ and CD127^+^ KLRG1^-^ T_MPE_. ***p < 0.001; ****p < 0.0001 (two-way ANOVA). **(C)** Percentage of splenic CD127^-^ KLRG1^+^ T_SLE_ and CD127^+^ KLRG1^-^ T_MPE_. **(D)** The total number of GP33-specific splenic T_SLE_ and T_MPE_ was calculated based on viable splenocyte counts. Mean ± SEM from two independent experiments with three to four mice per group. *p < 0.05; **p < 0.005 (Mann-Whitney).

We further investigated the impact of the absence of IL4I1 on T-cell differentiation by analyzing the cell surface expression of KLRG1 and CD127 by GP33-specific CD8^+^ T cells during the various phases of LCMV infection (**Figure 2**). These markers define two main populations: short-lived KLRG1^+^ CD127^-^ effector T cells (T_SLE_), which correspond to terminally differentiated highly cytotoxic cells, and KLRG1^-^ CD127^+^ memory precursor effector T cells (T_MPE_), which represent the main source of long-lived recirculating memory cells. IL4I1^-/-^ mice developed significantly fewer circulating T_SLE_ from day 6 to day 35 (**Figures 2A, B**, upper panels), whereas CD127 was up-regulated at a similar rate as in WT mice during the expansion phase. However, the T_MPE_ population of IL4I1^-/-^ mice progressively increased from the end of the contraction phase (**Figure 2B**, lower panel). At the beginning of the memory phase, there was a marked enrichment of circulating T_MPE_ in IL4I1^-/-^ mice, with reciprocal diminution of the T_SLE_ population (**Figure 2A**, representative data). We also observed an accumulation of virus-specific T_MPE_ in the spleens of IL4I1^-/-^ mice, both in terms of proportion and absolute number (**Figures 2C, D** **)**. Thus, IL4I1 affects the differentiation of CD8^+^ effector T cells, leading to preferential development of terminally-differentiated effector cells at the expense of cells with long-lived memory potential.

### Modulation of CD8^+^ T-Cell Differentiation by IL4I1 Is T-Cell Extrinsic

**Figure 3:**
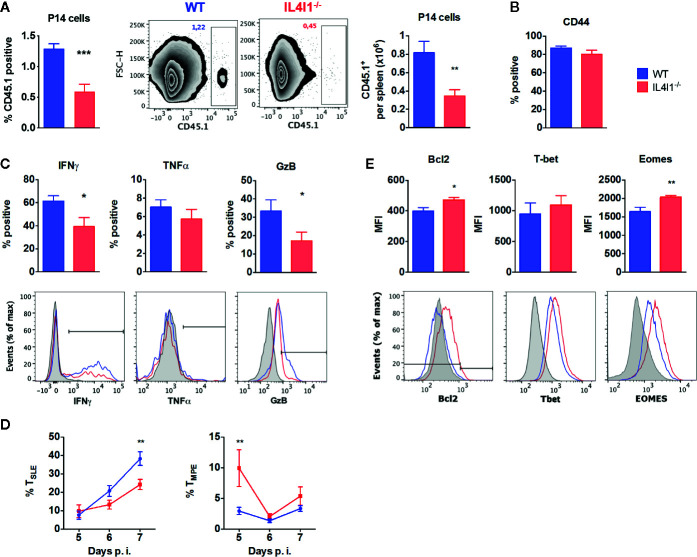
GP33-specific TCR-transgenic P14 CD8 T cells phenocopy the response of the endogenous repertoire when transferred into IL4I1^-/-^ hosts. CD8 T cells from CD45.1^+^ P14 mice were adoptively transferred into CD45.2^+^ congenic WT (blue) and IL4I1^-/-^ (red) mice one day before i.v. infection with LCMV. The expression of various markers was analyzed on CD45.1^+^ splenic P14 cells by flow cytometry **(A)**. Percentage and number of CD45.1^+^ CD8^+^ P14 cells in viable lymphocytes at day 5, with representative FSC/CD45.1 dot-plots **(B)**. Expression of CD44 by P14 cells at day 5 **(C)**. Percentage of CD44^+^ P14 cells expressing IFN*γ* and TNFα at day 5 and GzB at day 6. The lower monoparametric histograms show representative results with the negative control in gray (NP50-stimulated cells for IFN*γ* and TNFα, FMO for GzB) **(D)**. Percentage of CD127^-^ KLRG1^+^ T_SLE_ and CD127^+^ KLRG1^-^ T_MPE_ in P14 cells from days 5 to 7. Mean ± SEM from two to four independent experiments with three to four mice per group **(E)**. Mean fluorescence intensity (MFI) for Bcl2, T-bet, and Eomes in CD44^+^ P14 cells at day 7. The lower monoparametric histograms show representative results with the negative control in gray (isotype immunoglobulin for Bcl2; expression on CD8^+^ CD44^-^ cells for T-Bet and Eomes). *p < 0.05, **p < 0.005, ***p < 0.001 (Mann-Whitney, except **(E)**: Student t test).

We next transferred CD8^+^ T cells from P14 CD45.1^+^ congenic mice into WT or IL4I1^-/-^ mice (CD45.2^+^) to determine whether the difference observed between the response of WT and IL4I1^-/-^ mice could be due to intrinsic expression of IL4I1 in T cells (not described for CD8^+^ T cells) or modifications of the T-cell repertoire in IL4I1^-/-^ mice. These cells are IL4I1-competent and express a GP33-specific clonal TCR. Only 5,000 P14 cells were injected to minimize the differentiation bias introduced by adoptive transfer of a clonal population (20, 21). Five days post-infection, the P14 cells detected in WT and IL4I1^-/-^ mice expressed similar levels of the activation marker CD44, but their expansion was markedly lower in IL4I1^-/-^ mice (**Figures 3A, B** **)**.

As observed for the endogenous T-cell repertoire, P14 cells collected from IL4I1^-/-^ mice during the expansion phase (days 5–7) displayed a decreased capacity to produce IFN*γ* and granzyme B (**Figure 3C**). Accordingly, a lower percentage of P14 T_SLE_ was detected in IL4I1^-/-^ mice at day 7 (**Figure 3D**). We finally examined the expression of molecules that play an active role in the acquisition of effector functions and the formation of CD8 T-cell memory. At day 7 post-infection, P14 cells that had not been exposed to IL4I1 expressed higher levels of Eomes and Bcl2 than P14 cells transferred into WT mice, but there was no significant difference in T-bet levels (**Figure 3E**).

Thus, the absence of IL4I1 limits the early expansion and effector differentiation of transferred antigen-specific CD8 T cells and favors the development of cells with enhanced memory potential, as observed for endogenous polyclonal T cells. These results suggest a role for cells of the host at the time of antigen priming.

### Intrinsic Modifications of DC Populations by IL4I1 Expression Do Not Explain Differences in CD8^+^ T-Cell Priming

The induction of the CD8^+^ T-cell response is modulated by signals exchanged between DCs and CD8^+^ T cells during synaptic contact and by stimulating or inhibiting external cues, such as innate cytokines. The innate immune response to LCMV is dominated by the production of type I IFNs, which conditions DCs for optimal CD8^+^ T-cell activation and restrains Treg activation, as well as directly enhancing CD8^+^ T-cell expansion and differentiation (22, 23). However, there were no significant differences in the plasma concentration of IFNα between WT and IL4I1^-/-^ mice during the first 3 days after LCMV infection (**Figure S1**). The plasma concentration of five other innate cytokines (IL-6, IL12p70, TNFα, GM-CSF, and IL-1β) and the chemokine IP10 was also similar for both groups at days 1 and 2, with GM-CSF and IL-1β being barely detectable (**Figure S1B**). The percentage of NK cells (**Figure S1C**) and CD4^+^ T cells (**Figure S1D**) in the blood and spleen was identical at steady state for the two groups and splenic CD4^+^ T cells followed similar kinetics from day 2 to day 8 post-infection (**Figure S1D**, right graph). Finally, there was also no difference in the percentage of total and activated Treg at day 6 between infected IL4I1^-/-^ and WT mice (**Figure S1E**).

**Figure 4:**
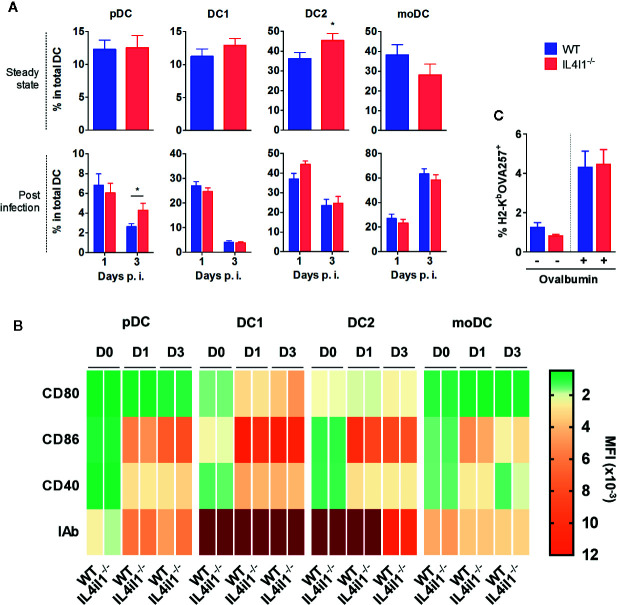
Repartition, maturation phenotype, and antigen-presenting capacity of splenic DC populations from IL4I1^-/-^ and WT mice **(A)**. DC subsets from WT (blue) and IL4I1^-/-^ mice (red) were analyzed by flow cytometry, according to the gating strategy presented in **Figures S2 **and** S4**, at steady state and days 1 and 3 p.i., respectively. The percentage of plasmacytoid DCs (pDC), conventional type 1 (DC1) and type 2 (DC2) DCs, and monocyte-derived DCs (moDC) was calculated in the total DC Boolean gate **(B)**. Mean fluorescence intensity of CD80, CD86, CD40, and IA^b^ on each subset at steady state (D0) and days 1 (D1) and 3 (D3) p.i. are presented as a heat map. The corresponding values are plotted in **Figure S3** (D0) and S5 (D1 and D3). Mean (± SEM where applicable) from six (steady state) and three experiments (p.i.) with two to three mice per group **(C)**. Total DCs enriched by negative magnetic cell sorting were cultured in the presence of ovalbumin 2 h before measuring presentation of the OVA257 peptide by flow cytometry using an antibody specific for H2-K^b^OVA257 complexes. Mean ± SEM from two cell sorting experiments with four independent tests per condition. *p < 0.05 (Mann-Whitney).

Thus, we hypothesized that DCs have a direct role in modulating the activation of CD8 T cells. We first analyzed splenic DC subpopulations of naïve mice, searching for intrinsic modifications of their number or phenotype in IL4I1^-/-^ mice (**Figure S2A**). We analyzed four DC subsets: type 1 conventional DCs (DC1, CD11c^+^ CD8α^+^), type 2 conventional DCs (DC2, CD11c^+^ CD11b^+^), plasmacytoid DCs (pDC, PDCA1^+^ SIGLECH^+^), and monocyte-derived DCs (moDC, CD11b^+^ CD11c^-^ IAb^+^). We observed no significant difference in the proportions of these DCs, except for a minor increase in the percentage of DC2 in IL4I1^-/-^ mice (**Figure 4A**, upper histograms). There were also no differences in the levels of the maturation markers CD80, CD86, CD40, and IA^b^ (**Figures 4B** and **S3**). As expected from the data of Montoya et al. (22), LCMV induced profound modifications of the percentages and phenotypes of splenic myeloid cells, including DC populations (**Figures 4**, **S4 and S5**). The changes were similar in WT and IL4I1^-/-^ mice, with the exception of a greater percentage of pDCs at day 3 in IL4I1^-/-^ mice.

We extended these results by performing transcriptomic analysis on sorted bulk uninfected CD11c^+^ populations from WT and IL4I1^-/-^ mice using a negative enrichment method to limit cell death and activation. NGS analysis (**Table S1**) showed that apart from *Il4i1*, 111 genes (77 protein-coding) were differentially expressed, among which none were expected to confer any advantage for T-cell priming or effector differentiation to WT DCs. In particular, comparative Gene Ontology (GO) analysis revealed no differences related to antigen processing and presentation, costimulation and negative regulation of the immune response, toll-like receptors, or cytokines and cytokine-mediated signaling pathways. A complete list of the GO terms, results for the 86 most highly expressed genes for these GO terms, and results for the GO term antigen processing and presentation are presented in **Figure S6**.

Finally, we examined whether variations in the antigen processing capacity of DCs can be induced by IL4I1. After incubating DCs with ovalbumin, we analyzed the cross-presentation of the OVA257 peptide using an antibody specific for the H2-K^b^OVA257 complex. WT and IL4I1^-/-^ DCs did not differ in their antigen presenting function (**Figure 4C**).

Overall, with the notable exception of IL4I1 itself, the T-cell activating capacity of DC populations from the two strains appeared to be similar, suggesting that IL4I1 has no major influence on their development or maturation. Therefore, we speculated that IL4I1 secreted by DCs directly modulates CD8 T-cell activation and differentiation. This effect may be mediated by the direct inhibition of TCR activation and synapse formation by IL4I1.

### Decreased T-Cell Activation Threshold in the Absence of IL4I1

**Figure 5:**
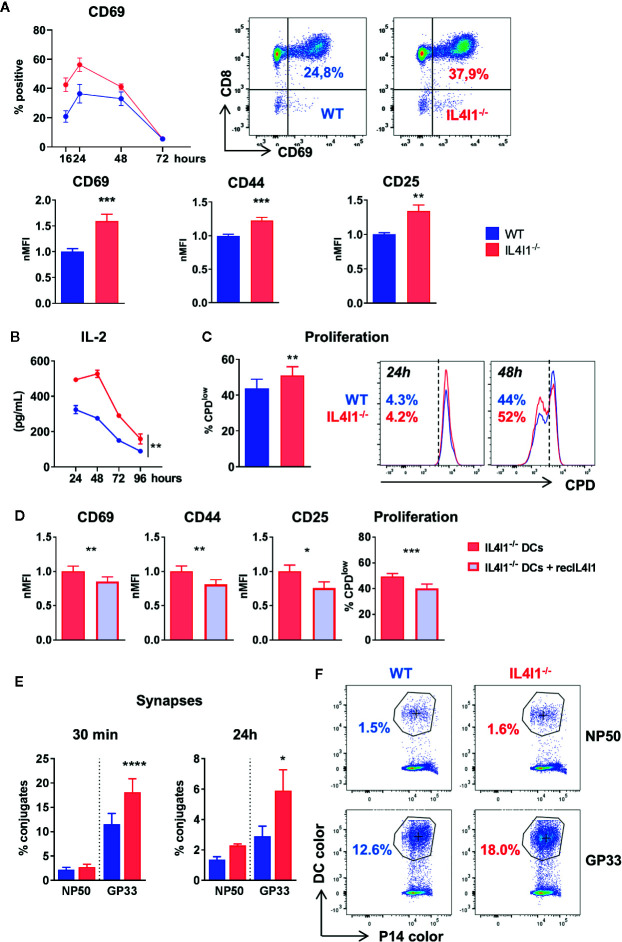
Stronger *in vitro* activation of P14 cells by IL4I1^-/-^ DCs. DCs from WT (blue) and IL4I1^-/-^ (red) mice were sorted negatively, pulsed with the GP33 peptide, and plated with sorted P14 CD8^+^ T cells at a 1:10 ratio. **(A)** Expression of surface activation markers measured by flow cytometry on P14 cells. Top graph and dot-plots: kinetics of CD69^+^ P14 cells (left; mean ± SEM from one representative experiment, with two tests per condition using DCs sorted from independent mice) and representative data at 16 h (middle and right). Bottom histograms: the MFI of CD69, CD44, and CD25 at 24 h was normalized to the mean results in cocultures with WT DCs (nMFI). Mean ± SEM of values from a minimum of 12 tests per condition obtained in six to eight independent experiments. **p < 0.005, ***p < 0.001 (Paired t-test). **(B)** Kinetics of IL-2 production in the cocultures from one representative experiment (n = 2 tests per condition). **p < 0.005 (two-way ANOVA). **(C)** Proliferating fraction of P14 cells at 48 h measured by flow cytometry using cell proliferation dye (CPD). Mean ± SEM from four experiments in duplicate (left) and representative data of a proliferation assay at 24 and 48 h (right). **(D)** Activation and proliferation of P14 cells measured as in A and C after coculture with IL4I1^-/-^ DCs in the presence or absence of recombinant murine IL4I1 (recIL4I1). Mean ± SEM of eight tests from four independent experiments. *p < 0.05 **p < 0.005, ***p < 0.001 (Paired t-test). **(E)** DCs and P14 cells were stained with different cell dyes. The percentage of P14 cells forming conjugates with DCs pulsed with GP33 or the control NP50 peptide were measured by flow cytometry at 30 min and 24 h after starting the coculture. Mean ± SEM of nine and three independent experiments at 30 min and 24 h, respectively, with one to three tests per condition. *p < 0.05, ****p < 0.0001 (Paired t-test). **(F)** Representative analysis of P14 conjugates at 30 min. Left: WT DCs, right: IL4I1^-/-^ DCs. The circled “bicolor” population corresponds to conjugates.

We sought to examine the effect of murine IL4I1 secreted by DCs on the activation of antigen-specific T cells. WT and IL4I1^-/-^ DC populations pulsed with GP33 were used to prime CD8^+^ naïve P14 T cells in culture. The expression of activation markers (**Figure 5A**), production of IL-2 (**Figure 5B**), and proliferation (**Figure 5C**) of antigen-stimulated P14 cells were reduced by the presence of IL4I1 naturally secreted by WT DCs. Similar inhibition was observed after exogenous addition of recombinant mouse IL4I1 (recIL4I1) in cocultures of P14 cells with IL4I1^-/-^ DCs (**Figure 5D**). This firmly demonstrates that IL4I1 is directly responsible for CD8 T-cell inhibition by DCs.

We recently showed that human IL4I1 diminishes immune synapse formation of polyclonally stimulated human T cells using the THP1 cell line as a source of antigen presenting cells. Here, we incubated stained P14 cells with WT and IL4I1^-/-^ DCs pulsed with GP33 to measure the formation of DC-T cell conjugates by flow cytometry. DCs pulsed with the influenza-derived NP50 peptide were used to measure non-specific conjugates. The DCs were either labeled with the same tracer and cultured separately or labeled with distinct tracers and mixed. P14 cells formed significantly fewer specific complexes with WT DCs than IL4I1^-/-^ DCs, both at 30 min and 24 h, even though the number of synapses had decreased by approximately 70% at 24 h (**Figures 5E, F** **)**. This suggests that IL4I1 diminishes synapse formation in the mouse.

We reasoned that *in vivo* competition for antigen access between naive T cells may be enhanced by the presence of IL4I1, facilitating the activation of T cells with the highest TCR affinity, such as P14. We tested this hypothesis *in vitro* by adding polyclonal, naïve, WT CD8 T cells to the P14-DC co-cultures. A 1:5 ratio of P14 cells was used to allow monitoring of the T-cell response at the same time points as those used for the control P14-DC co-cultures. Under these conditions, the P14 cells were outnumbered by T cells with poor to no affinity for the GP33-H2-D^b^ complexes, as high affinity for a specific epitope is a very rare event in the normal naive repertoire (24, 25).

**Figure 6:**
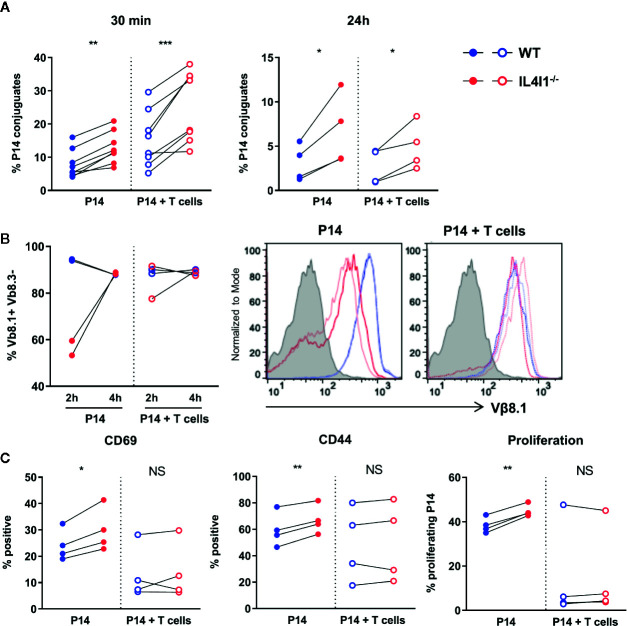
Activation of P14 clonal T cells by DCs in the presence of naïve polyclonal T cells. DCs from WT (blue) and IL4I1^-/-^ (red) mice presenting GP33 were obtained as in **Figure 5** and used to activate sorted P14 CD8^+^ T cells at a 1:10 ratio in the presence (empty circles) or absence (plain circles) of polyclonal T CD8^+^ cells sorted from a congenic naïve WT mouse (P14 cell:total T cell ratio = 1:5). **(A)** The percentage of P14 cells forming conjugates with GP33-pulsed DCs was measured as in **Figure 5E** at 30 min and 24 h. Individual results from, four and two independent experiments performed in duplicate, respectively. Pairing was based on the purity of the sorted DC populations. **(B)** Surface expression of the TCR Vβ8.1 chain by P14 cells was measured 2 and 4 h after activation in one experiment from panel A (2 tests per condition and time point). Left: percentage of Vβ8.1^+^ Vβ8.3^-^ cells in CD8^+^ T cells; right: corresponding raw flow cytometry data (grey histogram: negative control). **(C)** The percentage of CD45.1^+^ P14 cells expressing CD69 and CD44 and that of proliferating P14 cells were measured as in **Figure 5**. Results from four independent experiments in duplicate. NS, not significant; *p < 0.05, **p < 0.005, ***p < 0.001 (Paired t-test).

Unexpectedly, the addition of polyclonal T cells did not reduce, but instead increased the formation of immune synapses by P14 cells at 30 min by at least two fold. This was observed for both WT and IL4I1^-/-^ DCs (**Figure 6A**). However, at 24 h, the presence of polyclonal T cells no longer had an influence on the number of synapses and IL4I1^-/-^ DCs still formed a higher percentage of conjugates than WT DCs. Experiments conducted using a higher dilution of the P14 clone by the naïve polyclonal population (ratio 1:10 and 1:20) gave similar results (data not shown). Thus, the absence of IL4I1 facilitates synapse formation, even when low-ratio clonal competition is introduced. For comparison, the estimated P14:T-cell ratio in mice transferred with 5,000 P14 cells (as in **Figure 3**) is estimated to be between 1:4x10^3^ and 1:8x10^3^ (26), thus involving much more stringent clonal competition.

We analyzed surface TCR expression (Vβ8.1 chain) by P14 cells 2 and 4 h post-activation. At 2 h, we only observed downmodulation of the TCR for P14 cells cultured alone with IL4I1^-/-^ DCs. In particular, we detected no TCR downmodulation for P14 cells cultured with IL4I1^-/-^ DCs in the presence of polyclonal T cells (**Figure 6B**). TCR expression returned to basal levels at 4 h for all conditions. These results suggest that clonal competition can limit TCR activation in the absence of IL4I1.

We next examined the expression of activation markers and the proliferation of “T-cell diluted” P14 cells. The presence of polyclonal T cells mostly reduced P14 cell activation and proliferation, irrespective of the presence of IL4I1 (**Figure 6C**, compare full circles with empty circles). In this setting, the greater response of P14 cells cultured with IL4I1^-/-^ DCs was variable and the results from the comparison of WT and IL4I1^-/-^ conditions were no longer significant (**Figure 6C**, right part of the graphs).

Thus, P14 cells are activated better by antigen-presenting IL4I1^-/-^ DCs, particularly when they are not competing with naïve polyclonal T cells, indicating a decreased activation threshold in the absence of IL4I1.

### The Antigen-Responding T-Cell Repertoire Is Modulated by IL4I1

**Figure 7:**
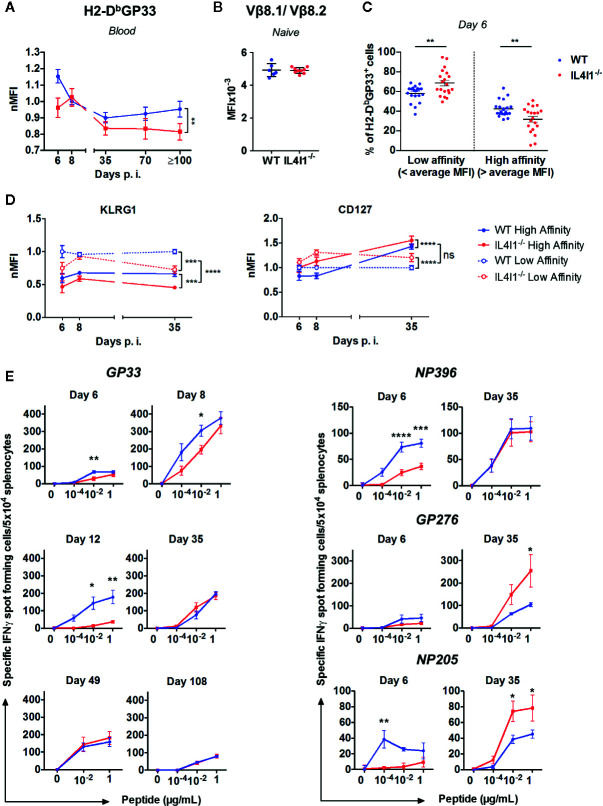
IL4I1-induced differences in the affinity and avidity of the LCMV-specific CD8^+^ T cell repertoire. WT (blue) and IL4I1^-/-^ (red) mice were infected i.v. with LCMV on day 0 **(A)**. TCR affinity estimated by tetramer binding. The MFI of H2-D^b^GP33 on H2-D^b^GP33^+^ CD8^+^ T lymphocytes was determined in blood samples from day 6 to ≥ day 100 p.i. and normalized against the average MFI obtained at day 8 in the WT mouse group for each experiment. Results were obtained from at least three independent experiments (day 6, n= 6; day 8, n=13; day 35, n=7, day 70, n=3, day 100, n=3) with three to four mice per group. **p < 0.005 (two-way ANOVA) **(B)**. Comparison of TCR Vβ8.1/Vβ8.2 levels (MFI) in uninfected WT and IL4I1^-/-^ mice **(C)**. Circulating high-affinity and low-affinity H2-D^b^GP33^+^ CD8^+^ T cell subsets on day 6 were segregated by the respective average H2-D^b^GP33 MFI in WT and IL4I1^-/-^ mice. **p < 0.005 (Student t test) **(D)**. High-affinity (plain line) and low-affinity (dotted line) GP33-specific cells, as determined in panel C, were analyzed for their KLRG1 and CD127 levels (MFI were normalized at each time point to the WT low-affinity results) at days 6, 8, and 35 p.i. Results from 6 to 12 independent experiments (≥ 20 mice per time point). NS: not significant, ***p < 0.001, ****p < 0.0001 (two-way ANOVA) **(E)**. Evolution of the functional avidity of CD8^+^ T cells responding to the immunodominant peptides GP33 (left) and NP396, GP276, and NP205 (right) assessed by ELISPOT-IFN*γ* with graded peptide dilutions. Results from one to four independent experiments with 3 (day 12) to 12 (day 8) mice tested per group. *p < 0.05, **p < 0.005, ****p < 0.0001 (two-way ANOVA with Sidak’s multiple comparison). All data are expressed as the mean ± SEM.

Our results suggest that IL4I1 modifies *in vivo* clonal competition. We tested this hypothesis by measuring the structural avidity of circulating CD8^+^ T cells specific for H2-D^b^-GP33 complexes and comparing the phenotype of low and high avidity cells in LCMV infected IL4I1^-/-^ and WT mice. The normalized mean fluorescence intensity (nMFI) of H2-D^b^GP33 tetramer binding was significantly higher in WT mice from day 6 to the early and late memory phase, except at the peak (day 8) of the response (**Figure 7A**) and was not due to higher expression of the TCR (**Figure 7B**). These results indicate that the CD8^+^ T-cell repertoire elicited by GP33 was of higher affinity when IL4I1 was present.

We next divided tetramer-positive CD8^+^ T cells into low and high-affinity binders using the average MFI measured in WT and IL4I1^-/-^ mice from each experiment as a cut-off. As expected, low-affinity binders were more abundant at day 6 in IL4I1^-/-^ mice, whereas high-affinity binders were more abundant in WT mice (**Figure 7C**). We quantified the expression of KLRG1 and CD127 in each subpopulation at days 6, 8, and 35 post-infection (**Figure 7D**). Low-affinity CD8 T cells expressed higher levels of KLRG1 at all time points, indicating that they were in a more advanced stage of effector differentiation than the high-affinity binders. Consistent with the data shown in **Figure 2**, the absence of IL4I1 resulted in lower KLRG1 expression, but this difference disappeared by the peak of the response (day 8). Conversely, the absence of IL4I1 was associated with a higher level of CD127, both on high- and low- affinity CD8 T cells. High-affinity CD8 T cells expressed higher levels of CD127 than their low-affinity counterpart only at day 35, at the beginning of the memory phase. Overall, the expansion of low-affinity responding T cells is initially favored in the absence of IL4I1, but these cells preferentially adopt a memory phenotype.

We also measured the functional avidity of LCMV-specific splenic CD8^+^ T cells by testing the response to successive dilutions of the four immunodominant peptides presented in **Figure 1D** by ELISPOT-IFN*γ* (**Figure 7E**). The response to GP33 was tested at six time points from day 6 to day 108 and at three peptide concentrations. The capacity to secrete IFN*γ* at low GP33 doses was the highest at day 8 and was significantly higher in WT than IL4I1^-/-^ mice from day 6 to day 12. The functional avidity became similar for both strains with the development of the memory phase (days 35, 49, and 108). The response to NP396, GP276, and NP205 was tested at day 6 (expansion) and 35 (beginning of the memory phase). The results at day 6 were consistent with those in **Figure 1D**, with higher responses in WT mice at low peptide dilutions, except for GP276. Similar to GP33, the functional avidity increased between day 6 and day 35 for these three peptides. This increase mainly concerned CD8^+^ T cells from IL4I1^-/-^ mice, for which the response to the less immunodominant GP276 and NP205 became higher than that of WT mice at day 35. Thus, the hierarchy of peptide immunodominance was still present in WT mice at the beginning of the memory phase, with GP33 and NP396 triggering the strongest responses, whereas the responses of IL4I1^-/-^ mice to the four peptides were of roughly similar intensity.

## Discussion

Here, we describe the differentiation of CD8 T cells from the endogenous repertoire and from the transgenic P14 clone in IL4I1-competent and IL4I1-deficient mice after *in vivo* priming by an acutely cleared infection with LCMV. We show that the absence of IL4I1 is paradoxically associated with diminished expansion of T_SLE_ that produce effector cytokines and granzyme B. Conversely, the number of T cells specific for the immunodominant peptide GP33 is higher in the spleens of IL4I1^-/-^ mice at the beginning of the memory phase and is associated with a higher proportion of CD127^+^ KLRG1^-^ T_MPE_. Moreover, P14 cells express higher levels of the antiapoptotic molecule Bcl-2 and the memory-promoting transcription factor Eomes when transferred into IL4I1^-/-^ hosts.

The consequences on acute elimination of LCMV were limited in our model, as the splenic viral load at day 4 was only 65% higher in IL4I1^-/-^ mice and the virus was cleared by day 14. Moreover, differences in the viral load cannot explain the different level of expansion and divergent T_SLE_/T_MPE_ differentiation of GP33-specific CD8 T cells between WT and IL4I1^-/-^ mice. Indeed, higher antigen doses should have stimulated stronger T cell proliferation (27), whereas fewer proliferating CD8 T cells were detected in IL4I1^-/-^ mice (**Figure 1C**). In addition, in an adoptive transfer experiment in which mice received a high quantity of P14 CD8^+^ T cells (3x10^6^/mouse) before LCMV infection showed these cells to be more strongly activated in WT mice as soon as 24h after infection (CD25^+^ P14 T cells = 69.2% in IL4I1^-/-^ mice versus 48.2% in WT mice). Thus, the absence of IL4I1 modifies the balance of CD8 T-cell differentiation, favoring the development of cells with long-term memory potential at the expense of short-lived effector cells.

We sought the reason for the differences in CD8 T-cell differentiation observed after LCMV infection of IL4I1^-/-^ hosts. At steady state, IL4I1^-/-^ mice do not display visible signs of immune dysregulation, show normal organ morphology and histology, and the major immune cell populations (monocytes, polymorphonuclear cells, CD4^+^ and CD8^+^ T lymphocytes, Tregs, and NK cells) are present in the blood and lymphoid organs at frequencies similar to those in WT mice, with the exception of B cells [(5, 28), **Figures S1C, D**, and unpublished data]. Priming in the LCMV model is conditioned by inflammatory cytokines, in particular the substantial amount of IFNα produced in the infected splenic marginal zone (22, 23, 29, 30). However, we did not detect any difference in IFNα production nor that of five other cytokines or the chemokine IP10 during the first several days after infection. We also did not detect any difference in the expansion of CD4^+^ T cells or Tregs, two populations that are known to be affected by IL4I1 activity in humans (31) and that may modulate CD8^+^ T-cell priming (23, 32).

DCs produce IL4I1 (3) and are directly responsible for antigen presentation to naïve CD8 T cells (22, 32, 33). The reduced activation and delayed effector differentiation of CD8 T cells in IL4I1^-/-^ mice may have resulted from intrinsic defects of the priming capacity of DCs. However, our results showed the DC populations from IL4I1^-/-^ mice to be quantitatively similar to those of WT mice, with the exception of a slightly higher percentage of conventional DC2 at steady state and pDC at day 3 post-infection. They also showed no phenotypic differences nor differences in their antigen presenting capacity. Finally, transcriptomic analysis did not provide any arguments in favor of a diminished priming ability of IL4I1^-/-^ DCs.

The main difference between WT and IL4I1^-/-^ DCs was thus the production of IL4I1 itself, which is known to be secreted in immune synapses formed with T cells and to restrain the activation of signaling pathways downstream of the TCR in primary human T cells (2). Through the use of DCs to activate P14 clonal CD8 T cells *in vitro*, we confirmed that murine IL4I1 directly limits immune synapse formation, activation, and proliferation.

The CD8 T-cell outcome has been shown to depend on the intensity of the received signal in individual cells in several models (34). In particular, TCR affinity regulates the duration of immune synapses, the magnitude of the initial clonal burst, the migratory properties, and the time to effector cell differentiation, with low-affinity cells disengaging earlier from DCs to start the expansion and effector differentiation program (35, 36). An increase in the activation threshold, associated with the absence of the costimulatory molecule CD27, increases the affinity for the antigen of the elicited T-cell repertoire and T_SLE_ differentiation after infection (37). Conversely, we observed that the absence of the inhibitory enzyme IL4I1 enabled the priming of a CD8^+^ T-cell population of lower affinity for the immunodominant LCMV GP33 epitope and favored T_MPE_ differentiation. The preferential differentiation of low affinity CD8^+^ T cells into memory cells was recently confirmed in a study that identified high CD45RB expression as a new marker of both low TCR affinity and long-term survival (38). Moreover, ELISPOT-IFN*γ* analysis performed at day 6 and at the beginning of the memory phase showed that the functional avidity of T cells increased in IL4I1^-/-^ mice in response to GP33 and three other LCMV peptides. In particular, the response to the less immunodominant epitopes GP276 and NP205 was stronger in IL4I1^-/-^ mice, whereas the response to GP33 and NP366 reached the level observed in WT mice. Thus, by limiting synapse formation and enhancing the activation threshold, IL4I1 secreted by DCs facilitates the *in vivo* activation of cells with low-affinity TCRs and may enhance memory to less immunodominant antigens. We attempted to model the competition of a high-affinity T-cell clone with low-affinity T cells *in vitro* by activating P14 cells in the presence of naïve polyclonal CD8 T cells, but were unable to reproduce physiological ratios of high to low-affinity T cells because of technical limitations. Nevertheless, several of our results suggest that the absence of IL4I1 no longer provided an advantage for P14 cell activation when competing with other T cells.

As stated above, delayed proliferation of CD8^+^ T cells in IL4I1^-/-^ mice did not prevent LCMV clearance. The consequences may be more severe in chronic infections. However, we believe, on the contrary, that the absence of IL4I1 is favorable in situations of chronic antigen exposure, including cancer. Indeed, we previously reported that IL4I1 limits the expansion of anti-tumor cytotoxic CD8 T cells and accelerates the escape of mouse melanoma from immune control (5, 6). We also observed reduced development of transplanted EL4 tumors in the livers of IL4I1^-/-^ mice (**Figure S7**). Thus, antitumor CD8^+^ T cells primed in IL4I1^-/-^ hosts show better control of tumor cells. The phenotypic differences between WT and IL4I1^-/-^ mice observed early after priming in the LCMV model may be associated with enhanced antitumor properties, for example, an extended capacity to regenerate effector cells. Indeed, the *in vivo* eradication of cancer requires prolonged activation of the specific T-cell response. Concerning the mechanism that fosters effector T cell differentiation in the presence of IL4I1, it is tempting to speculate that it involves the production of H_2_O_2_. Indeed, reactive oxygen species have been shown to increase terminal differentiation, whereas antioxidants allow the generation of long-lived memory stem cells with enhanced antitumor properties (39). As we show that the absence of IL4I1 extends the repertoire to low-affinity T-cell clones, another explanation for the better control of tumors in IL4I1^-/-^ mice merits consideration. By favoring cross-reactivity, increased T cell diversity should be associated with better protection from antigen mutation or variation (40, 41). It was recently reported that CD8^+^ T-cell clones specific for the cytomegalovirus expand in successive phases during chronic infection in mice and humans, with the relative affinity decreasing over time due to sequential progression of the higher affinity clones towards senescence (42). These findings highlight the interest of low-affinity clones in maintaining a durable response to chronic stimulation.

**Figure 8:**
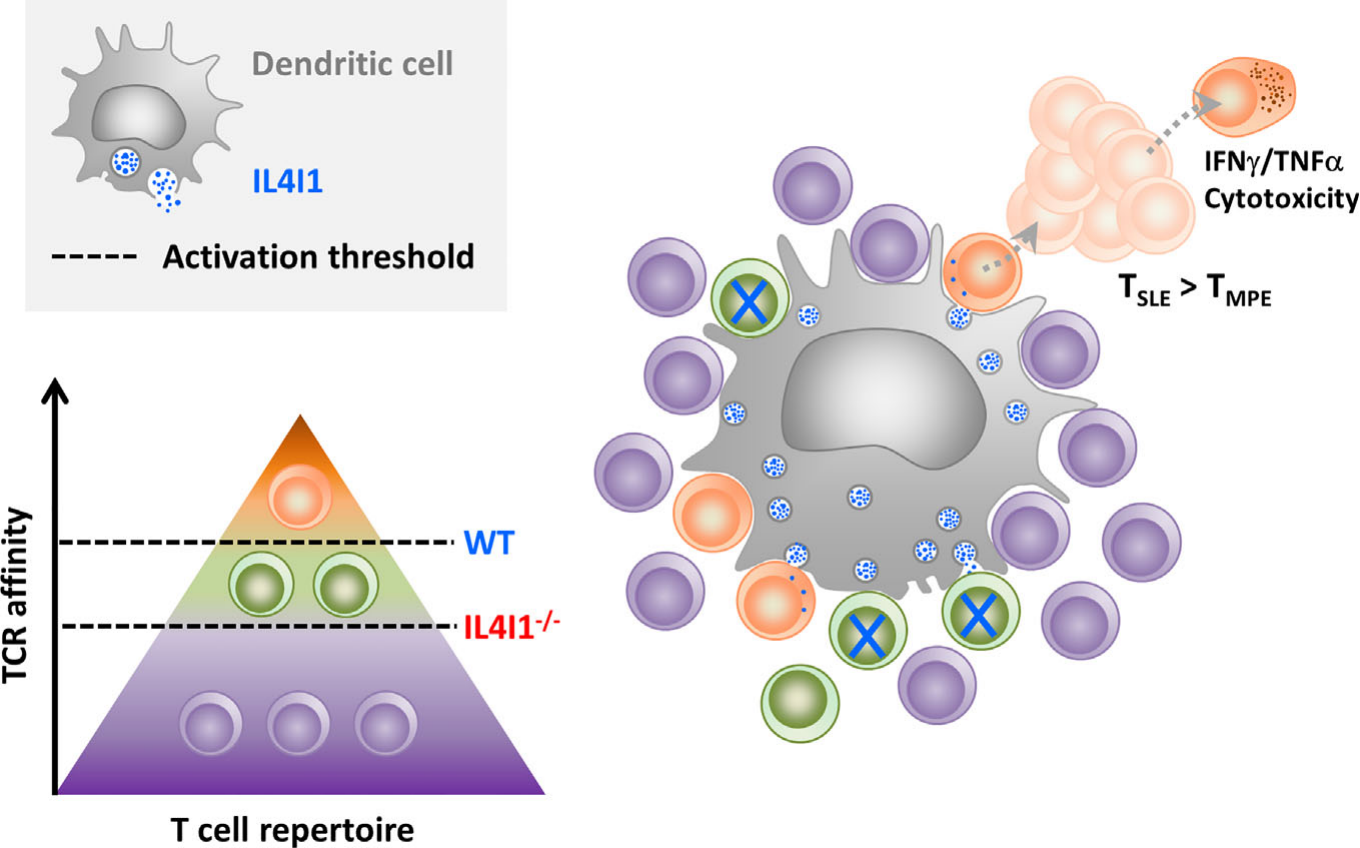
Hypothesis for CD8-T cell priming in the presence of inhibitory IL4I1. When secreted by DCs, the IL4I1 enzyme limits stable synapse formation and inhibits the activation of antigen-specific T cells. This enhances the activation threshold (dotted black line) of the polyclonal T cell population (symbolized by a pyramid), which mostly contains cells with poor or no affinity for the antigen (purple). In the context of a CD8^+^ T cell response to LCMV, this may favor the initial activation of high-affinity T cells (orange) at the expense of T cells with TCRs of lower affinity (green), as observed in WT mice. High-affinity clones proliferate rapidly and preferentially give rise to terminally-differentiated effector T cells. In contrast, low-affinity clones, which are higher in IL4I1^-/-^ mice, are more prone to acquire a T_MPE_ phenotype.

In conclusion, our *in vitro* and *in vivo* data suggest that IL4I1 enhances the capacity of high-affinity CD8 T cells to outcompete lower affinity T cells, thus accelerating their full activation, expansion, and T_SLE_ differentiation (**Figure 8**). On the other hand, the absence of IL4I1 facilitates T_MPE_ development, with consequences for the quality of long-term memory, potentially involved in the better resistance of IL4I1^-/-^ mice to tumor development. Our study demonstrates that, beyond solely inhibiting T-cell activation, IL4I1 can modulate the quality of the CD8^+^ T-cell response.

## Data Availability Statement

The datasets presented in this study can be found in online repositories. The names of the repository/repositories and accession number(s) can be found in the article/**Supplementary Material**.

## Ethics Statement

The animal study was reviewed and approved by Cometh Anses/ENVA/UPEC.

## Author Contributions

VM-F and FC conceived and supervised the experiments, analyzed the data, and wrote the manuscript. M-LP, AD, NS, and JG developed the methodology, performed the experiments, and analyzed the data. DM analyzed and interpreted the transcriptomic data. AL assisted in designing the *in vivo* experiments and interpreting the data (specific expertise with the mouse LCMV infection model). AP-B assisted in designing the experiments, analyzing the data, and writing the manuscript. JC helped in interpreting the data and edited the manuscript. All authors contributed to the article and approved the submitted version.

## Funding

This work was supported by INCA N°2018-155, FRM DEQ20160334875, and two grants from GEFLUC Paris-Ile de France.

## Conflict of Interest

The authors declare that the research was conducted in the absence of any commercial or financial relationships that could be construed as a potential conflict of interest.
