# Supplementary material for: IL4I1 Accelerates the Expansion of Effector CD8+ T Cells at the Expense of Memory Precursors by Increasing the Threshold of T-Cell Activation
Source: Front Immunol. 2020 Dec 4;11:600012. doi: 10.3389/fimmu.2020.600012 (PMC7746639; doi:10.3389/fimmu.2020.600012)
Supplement: Supplementary file 1 [file DataSheet_1.pdf]

Supplementary figures

Supplementary Figure 1

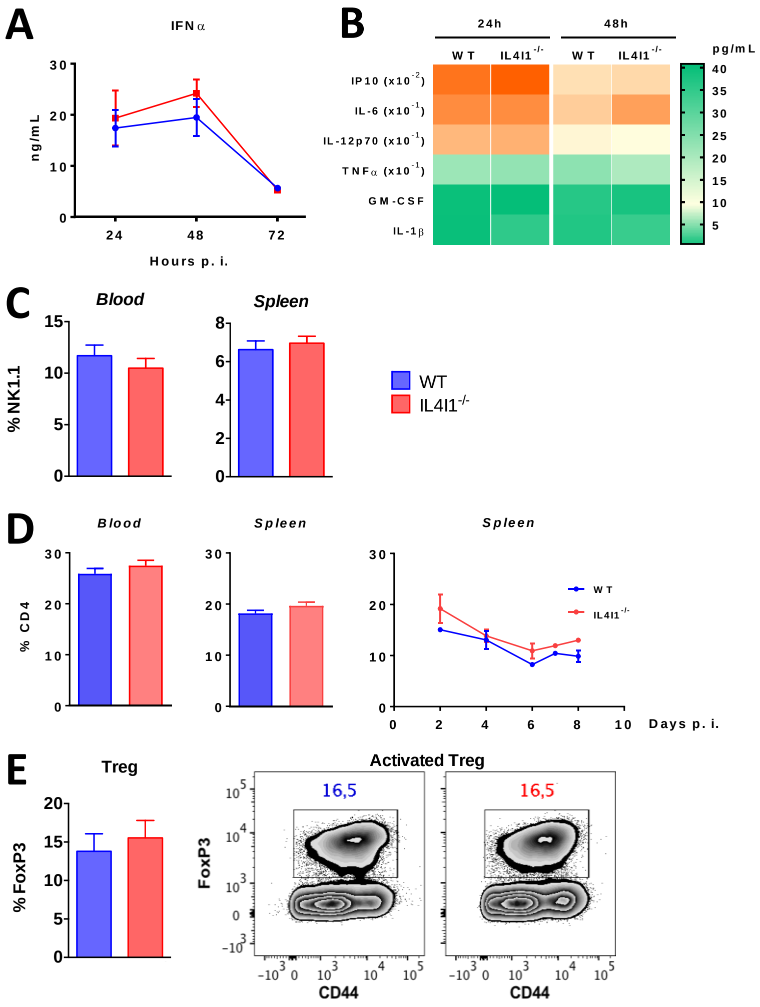

Parameters of the innate immune response, $CD4^+$ T cells and Tregs in WT and $IL4I1^{-/-}$ mice. (**A**) IFNα was measured by ELISA on plasmas collected 24h, 48h and 96h after infection with LCMV. (**B**) The chemokine IP10 and the cytokines IL-6, IL-12p70, TNFα, GM-CSF and IL-1β were measured on plasmas collected 24h and 48h p.i. using a multiplex immunodetection kit. Results in A and B are from 3 to 4 independent experiments with at least two mice per group . (**C**) The percentage of NK1.1 expressing cells was determined at steady state in the blood and spleen of WT and $IL4I1^{-/-}$ mice (n=6/group). (**D**) The percentage of $CD4^+$ cells was determined by flow cytometry in the blood and spleen of WT and $IL4I1^{-/-}$ mice at steady state (n=25/group) and in the spleen from day 2 to 8 following infection (3 independent experiments with 2 or 3 mice per group). (**E**) The percentage of $CD4^+$ $FoxP3^+$ Treg cells was determined by flow cytometry in the spleen at day 6 following infection (3 independent experiments with 3 mice per group). Day 6 activated $CD44^+$ Tregs cells were measured in one experiment (a representative result from one mouse per group is shown). Results are represented as mean ± SEM where applicable. Statistical analyses showed no difference between WT and $IL4I1^{-/-}$ mice.

Supplementary Figure 2

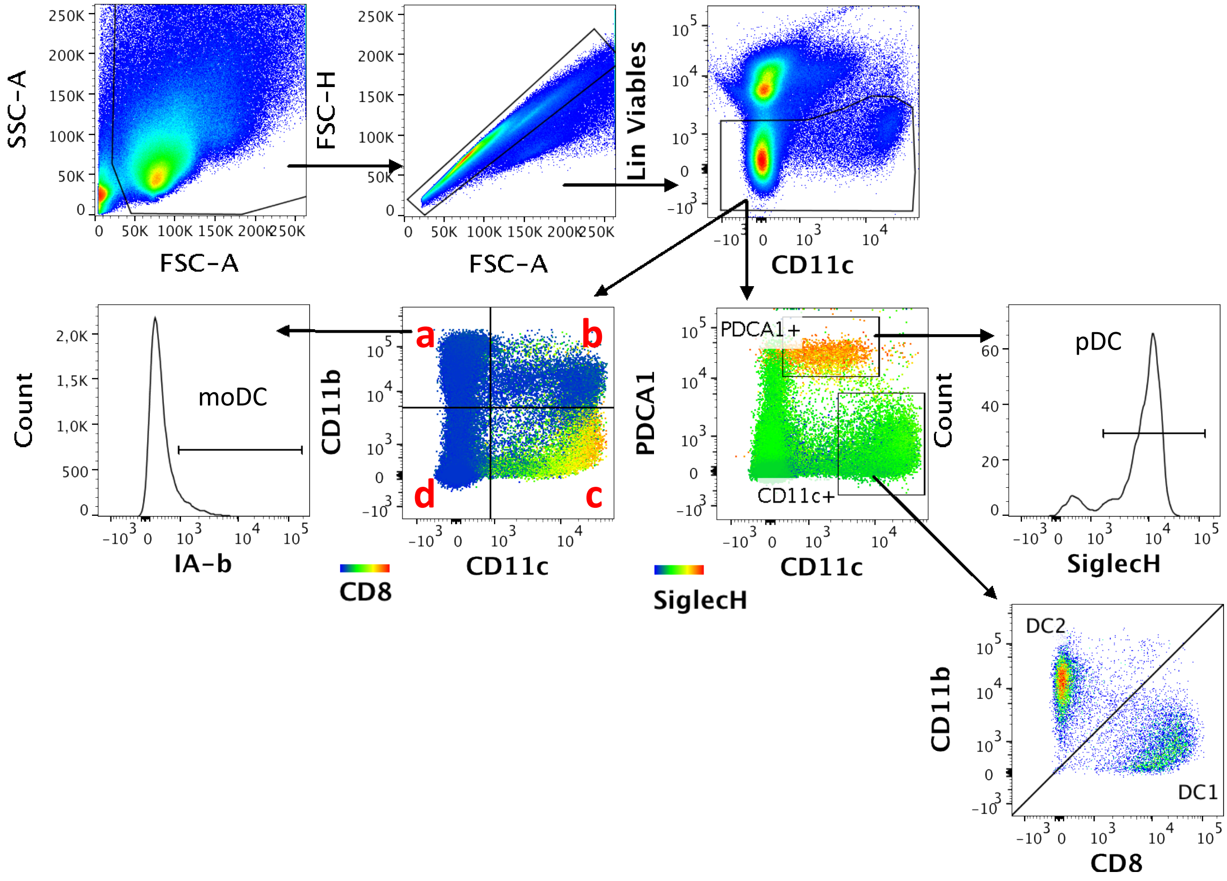

Identification of splenic DC subsets at steady state in WT and IL4I1$^{-/-}$ mice. The gating strategy is illustrated by representative results of a WT mouse. After exclusion of cell debris on the forward scatter (FSC)/side scatter (SSC) dot-plot, single cell events and linage (Lin) negative viable cells were successively selected (viability dye and lineage markers CD19, NK1.1 and CD3 signals are collected in the same fluorescence canal). pDCs were defined as PDCA1^{+} CD11c^{low} cells (SiglecH is also expressed). Conventional DCs were defined as PDCA1^{-} CD11c^{high} with DC1 and DC2 discriminated by expression of CD8α and CD11b. Monocyte-derived DC (moDCs) were defined as IA^{b}+ CD11b^{high} CD11c^{-} cells. Boolean gates including the four DC subsets were created to measure the percentages of each subset within the total DC population (Fig. 4).

Supplementary Figure 3

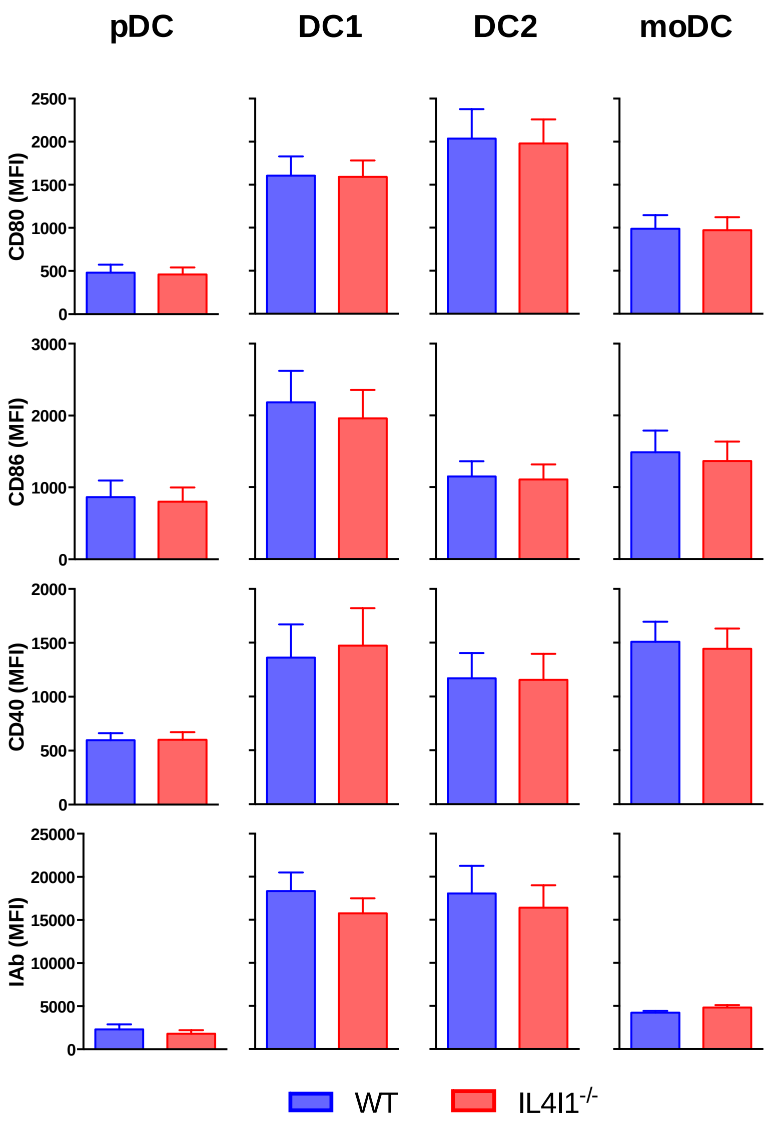

Maturation phenotype of steady state splenic DC subsets. Mean fluorescence intensity of CD80, CD86, CD40 and IA^b. Mean ± SEM from 5 experiments with 2 to 3 mice per group, corresponding to D0 data in the heat map presented in Fig. 4B. No significant difference between WT and $IL4I1^{-/-}$ mice was observed (Student's t test).

Supplementary Figure 4

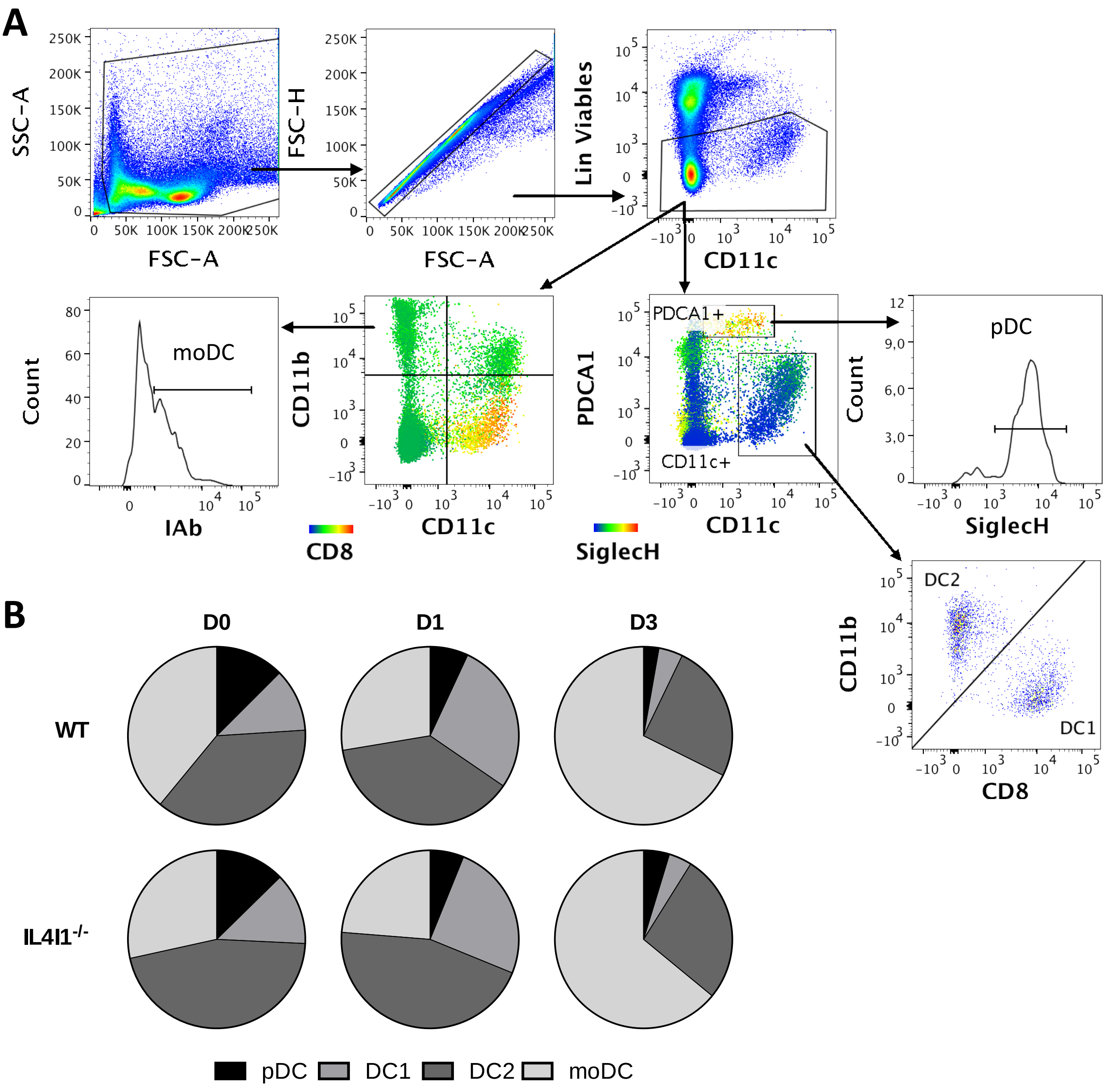

Identification of splenic DC subsets at day 1 and 3 after infection in WT and IL4I1$^{-/-}$ mice. (**A**) The gating strategy, which is similar as in Fig. S2A, is illustrated by representative results of a WT mouse at day 1 p.i. The four subsets — pDCs, conventional DC1 and DC2 and moDCs — are easily identified, but the intensity of expression of CD11b and IA^{b} are increased and the proportion of the subsets are modified. (**B**) Pie charts corresponding to data in Fig. 4A. This representation allows easy visualization of the modifications of DC repartition induced by infection.

Supplementary Figure 5

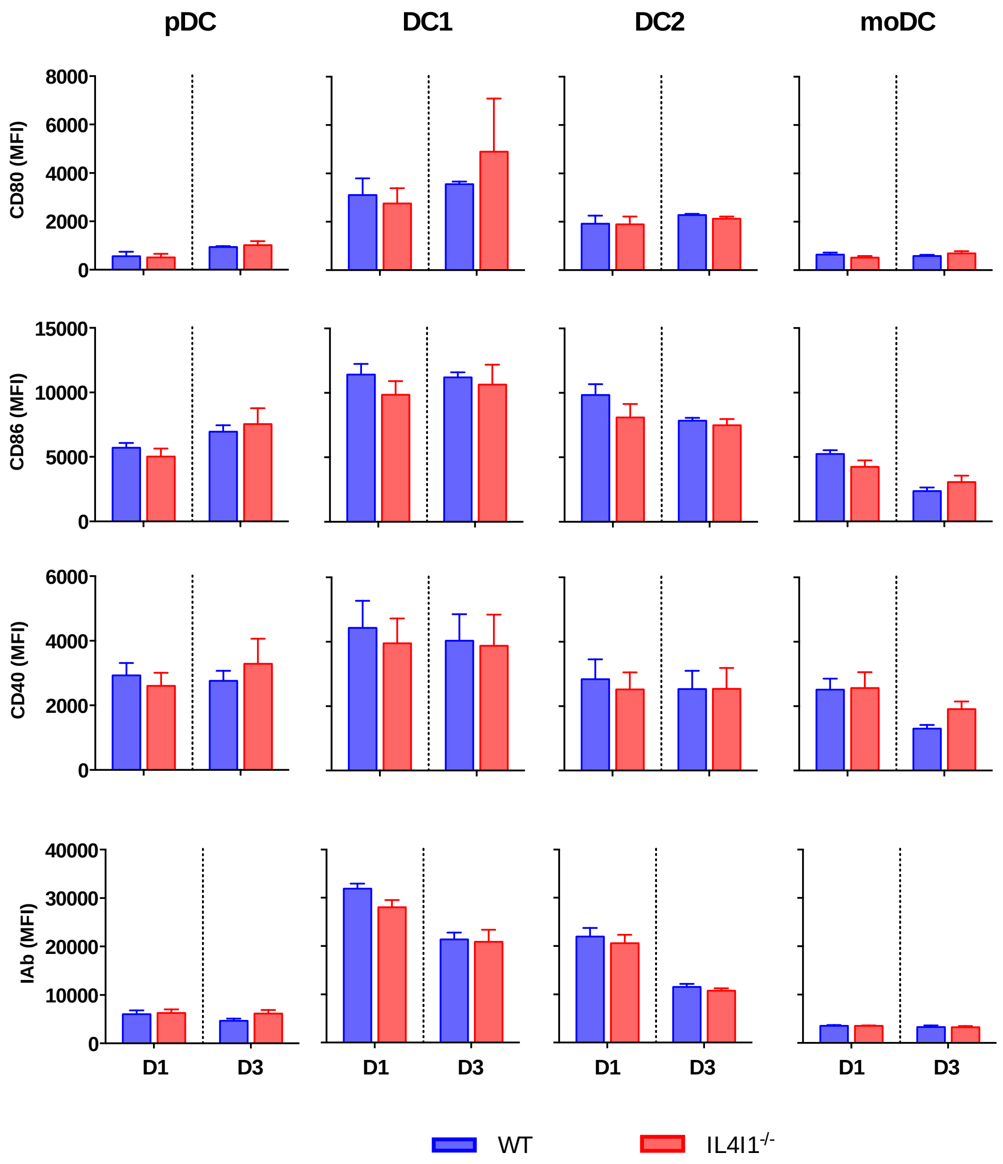

Maturation phenotype of splenic DC subsets at day 1 and 3 after infection. Mean fluorescence intensity of CD80, CD86, CD40 and IA^b. Mean ± SEM from 3 experiments with 2 to 3 mice per group, corresponding to D1 and D3 data in the heat map presented in Fig. 4B. No significant difference was observed (Student's t test).

Supplementary Figure 6

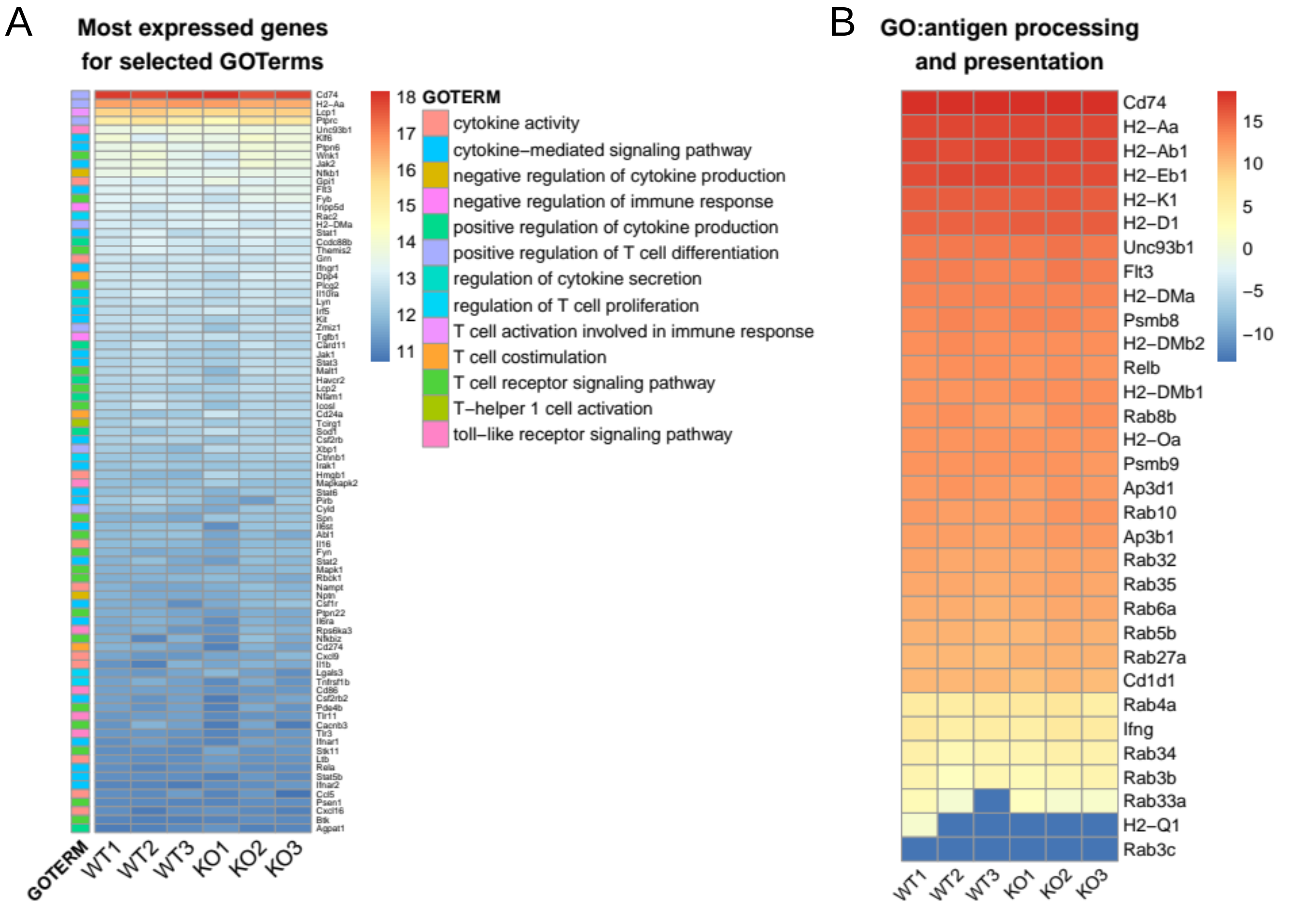

Results of the transcriptomic analysis of splenic DCs from WT and IL4I1$^{-/-}$ mice. (**A**) Heatmap of the 86 most expressed genes (in log2 Counts Per Millions (CPM)) with selected GO terms. (**B**) Heatmap of genes of the GO term “antigen processing and presentation” (GO:0019882). These two heatmaps show that the WT and KO mouse groups have similar expression profiles. Other GO terms analyzed (not represented) were related to costimulation (GO:0031296, GO:0035783, GO:1900280, GO:1900281, GO:2000525, GO:0031295), negative regulation of immune response (GO:0050777), toll-like receptors (GO:0034136, GO:0034140, GO:0034144, GO:0034148, GO:0034164, GO:0034122, GO:0032720, GO:0034181, GO:0034137, GO:0034141, GO:0034145, GO:0034157, GO:0034165, GO:0034123, GO:0034143, GO:0034163, GO:0034121, GO:0035665, GO:0034130, GO:0035354, GO:0034178, GO:0035663, GO:0034134, GO:0035355, GO:0034138, GO:0034142, GO:0034146, GO:0034150, GO:0034154, GO:0034158, GO:0034162, GO:0035325, GO:0002224, GO:0035669, GO:0035666), cytokines and cytokine-mediated signaling pathways (GO:0002368, GO:1990869, GO:0071345, GO:0005125, GO:0019955, GO:0042089, GO:0042107, GO:0001816, GO:0002367, GO:0002534, GO:0004896, GO:0005126, GO:0050663, GO:0002374, GO:0019221, GO:0002371, GO:0060302, GO:0042036, GO:0001818, GO:1900016, GO:0050710, GO:0002740, GO:0001960, GO:0002731, GO:0032764, GO:0002728, GO:0060761, GO:0002725, GO:2000552, GO:0042108, GO:0001819, GO:0002720, GO:1900017, GO:0050715, GO:0002741, GO:0001961, GO:0002732, GO:0060907, GO:0032765, GO:0002735, GO:0061081, GO:0002729, GO:0060760, GO:0002726, GO:2000406, GO:2000556, GO:2000553, GO:0060300, GO:0042035, GO:0001817, GO:0002718, GO:1900015, GO:0050707, GO:0002739, GO:0001959, GO:0002730, GO:0002724, GO:0042509, GO:0034097, GO:0002369, GO:0035744, GO:0035745).

Supplementary Figure 7

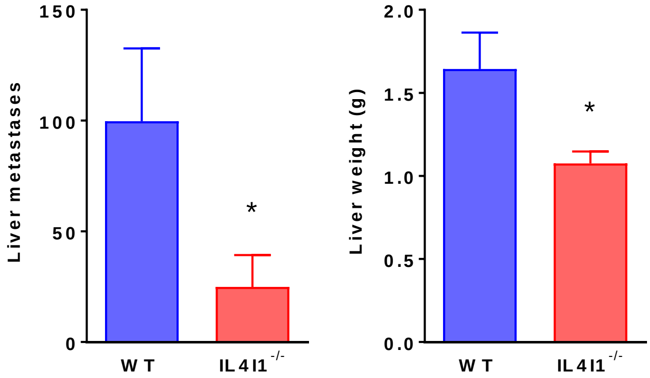

EL4 tumor growth in WT and IL4I1$^{-/-}$ mice. Mice were injected intravenously with $5x10^5$ cells of the EL4 lymphoma cell line at day 0. All the mice of the same experiment were simultaneously sacrificed between day 12 and 14 and the number of liver metastases and liver weight were measured. When the liver was massively invaded, the number of metastases was arbitrarily fixed at 250. Mean ± SEM of twelve mice per group from 3 experiments. *p<0.05 (Mann-Whitney).
